# Supplementary material for: Arguments for the biological and predictive relevance of the proportional recovery rule
Source: eLife. 2022 Oct 18;11:e80458. doi: 10.7554/eLife.80458 (PMC9648971; doi:10.7554/eLife.80458)
Supplement: Source data 1. [file elife-80458-data1.zip › prr_reproducibility/prop_recov_reproducibility.docx]

The proportional recovery rule redux

Arguments for biological and predictive relevance

Jeff Goldsmith, Robinson Kundert, Andreas Luft, Cathy Stinear, Winston Byblow, Angel Garcia de la Garza, Geert Kwakkel, Tomoko Kitago, John W. Krakauer

## Abstract

This document produces figures and tables included in the paper. The output is a Word document with all figures and their captions; high res versions of figures are exported to results/.

This document also includes some code chunks that don’t produce figures but are otherwise relevant to the paper. This includes empirical confirmations of some derived results, and the gap-statistic based analysis used to compare clustering results for Winters to a null of random recovery.

## CorPlot Base

## Simulated datasets


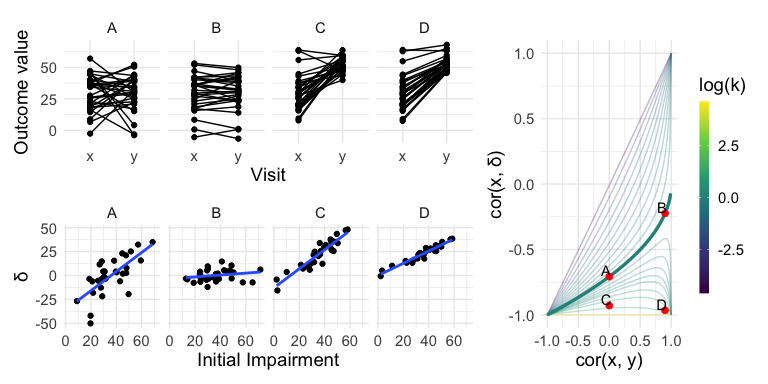


Figure 1: The left panels show four simulated datasets, labeled A, B, C, and D. In the top row, panels show outcome values and baseline (x) and follow-up (y). In the bottom row, panels show change (delta) against initial impairment (66 - x). The right panel shows a contour plot of Equation 1, with contours corresponding to values of the variance ratio k and the contour for k = 1 highlighted. Points on this surface show correlation values obtained for Dataset A through D.

## Not included: verification of computations

## Simulated data


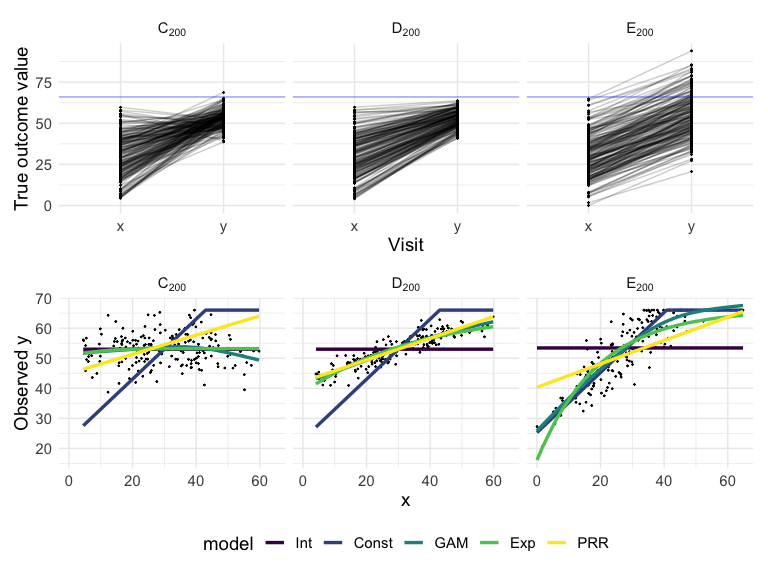


Figure 2: The top panels show three simulated datasets with true outcome values and baseline (x) and follow-up (y); a horizontal line indicates a ceiling on observed values. In the bottom row, panels show the observed (ceiled) value at follow-up against the baseline value.

## Cross Validation: Sim data


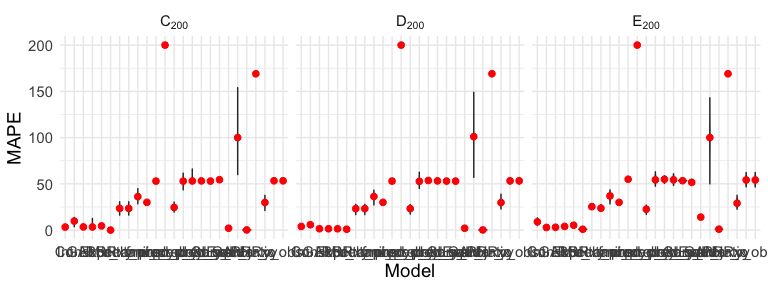


Figure 3: Each panel shows the distribution of median absolute prediction errors (MAPE) obtained using cross validation for each of three models. Panels correspond to the simulated datasets shown in Figure 2. Models compared are an intercept-only model, a generalized additive model, and the PRR.

## Reported datasets

## List of 1
## $ legend.position: chr "none"
## - attr(*, "class")= chr [1:2] "theme" "gg"
## - attr(*, "complete")= logi FALSE
## - attr(*, "validate")= logi TRUE


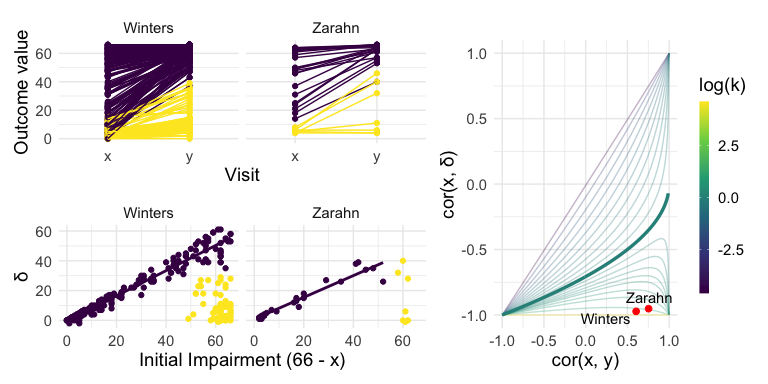


Figure 4: The left panels show three real datasets. In the top row, panels show outcome values and baseline (x) and follow-up (y); points are colored to indicate recoverers (purple) and non-recoverers (yellow) using the definitions from each paper describing the data. In the bottom row, panels show change (delta) against initial impairment (66 - x), again separating recoverers and non-recoverers. The right panel shows a contour plot of Equation 1, with contours corresponding to values of the variance ratio k and the contour for k = 1 highlighted. Points on this surface show correlation values obtained for the real datasets.

## Bootstrap: real data


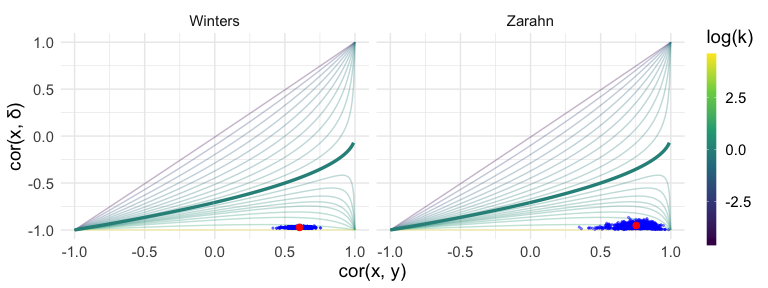


Figure 5: Each panel shows the results of the bootstrap procedure used to obtain inferences about the value of correlations and variance ratio. Red points are the values obtained for the full dataset, and blue points are values obtained in each of 1000 bootstrap samples; points are overlaid on the contour plot of Equation 1.

| name | k | cor_xy |
| --- | --- | --- |
| Winters | 0.06 [0.04, 0.09] | 0.6 [0.5, 0.7] |
| Zarahn | 0.13 [0.04, 0.24] | 0.75 [0.54, 0.91] |

## Cross validation: real data


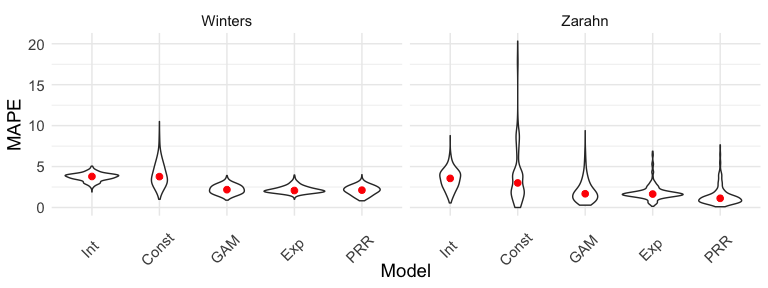


Figure 6: Each panel shows the distribution of median absolute prediction errors (MAPE) obtained using cross validation for each of three models. Panels correspond to the datasets shown in Figure 4. Models compared are an intercept-only model, a generalized additive model, and the PRR.

## MAPE: real data

| name | R2 |
| --- | --- |
| Winters | 36.4 |
| Zarahn | 56.4 |

## Clustering


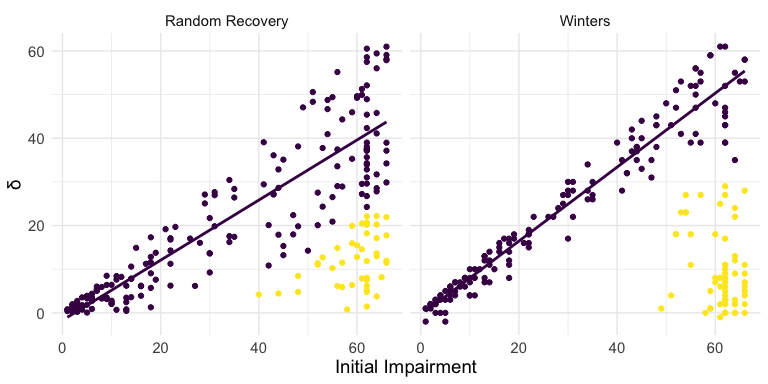


Figure 7: Both panels show change between baseline and follow-up against initial impairment (66 - baseline). Left panel shows data simulated under a ‘random recovery’ process, in which outcome values are drawn from a uniform distribution over the baseline value and ceiling. Right panels shows data from Winters et al.
