# Supplementary material for: Arguments for the biological and predictive relevance of the proportional recovery rule
Source: eLife. 2022 Oct 18;11:e80458. doi: 10.7554/eLife.80458 (PMC9648971; doi:10.7554/eLife.80458)
Supplement: Source data 1. [file elife-80458-data1.zip › prr_reproducibility/my_docx_styles.docx]

## Untitled

Jeff Goldsmith

7/1/2019

## R Markdown

This is an R Markdown document. Markdown is a simple formatting syntax for authoring HTML, PDF, and MS Word documents. For more details on using R Markdown see <http://rmarkdown.rstudio.com>.

When you click the **Knit** button a document will be generated that includes both content as well as the output of any embedded R code chunks within the document. You can embed an R code chunk like this:

summary(cars)

## speed dist
## Min. : 4.0 Min. : 2.00
## 1st Qu.:12.0 1st Qu.: 26.00
## Median :15.0 Median : 36.00
## Mean :15.4 Mean : 42.98
## 3rd Qu.:19.0 3rd Qu.: 56.00
## Max. :25.0 Max. :120.00

## Including Plots

You can also embed plots, for example:


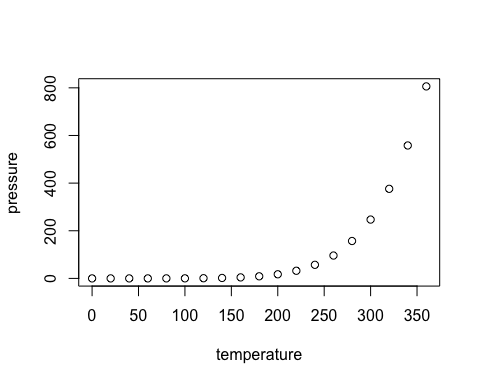


Figure

Note that the echo = FALSE parameter was added to the code chunk to prevent printing of the R code that generated the plot.
