# Supplementary figures and images for: Arguments for the biological and predictive relevance of the proportional recovery rule
Source: eLife. 2022 Oct 18;11:e80458. doi: 10.7554/eLife.80458 (PMC9648971; doi:10.7554/eLife.80458)

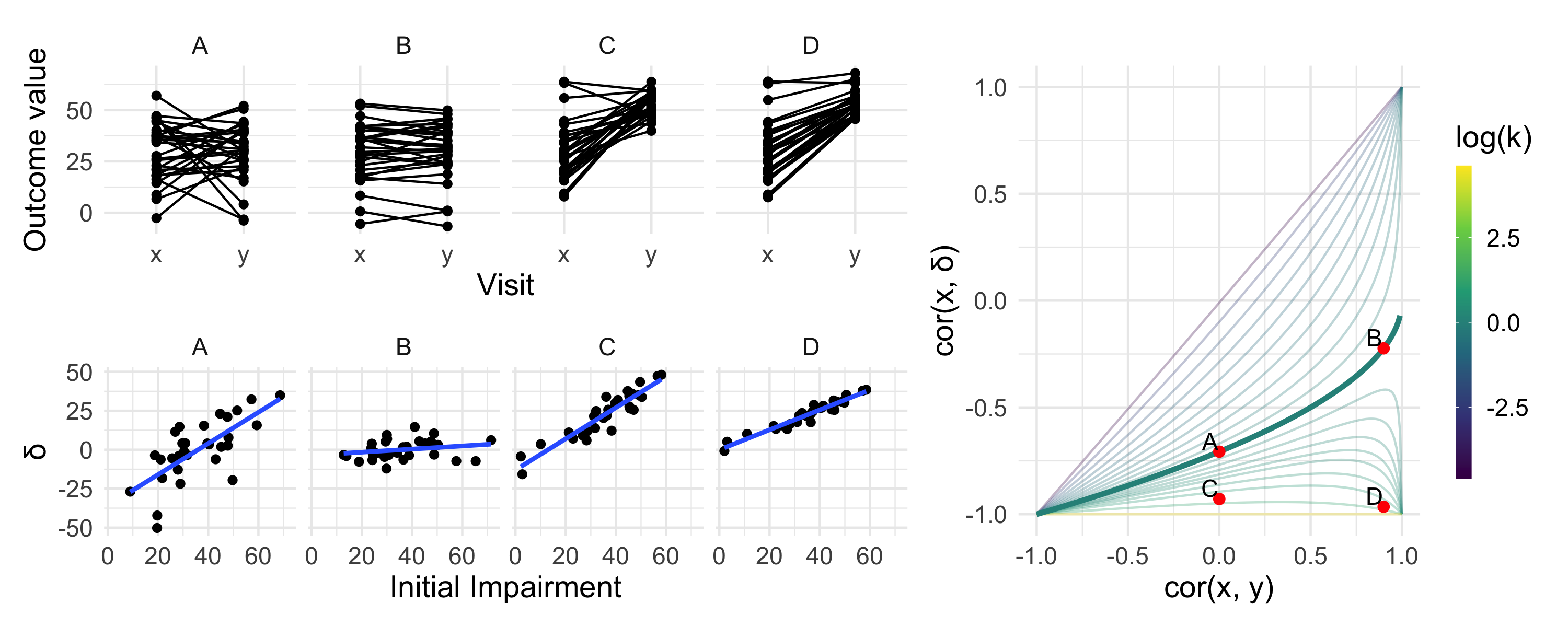

Supplement: Source data 1. [file elife-80458-data1.zip › prr_reproducibility/results/example_data.png]

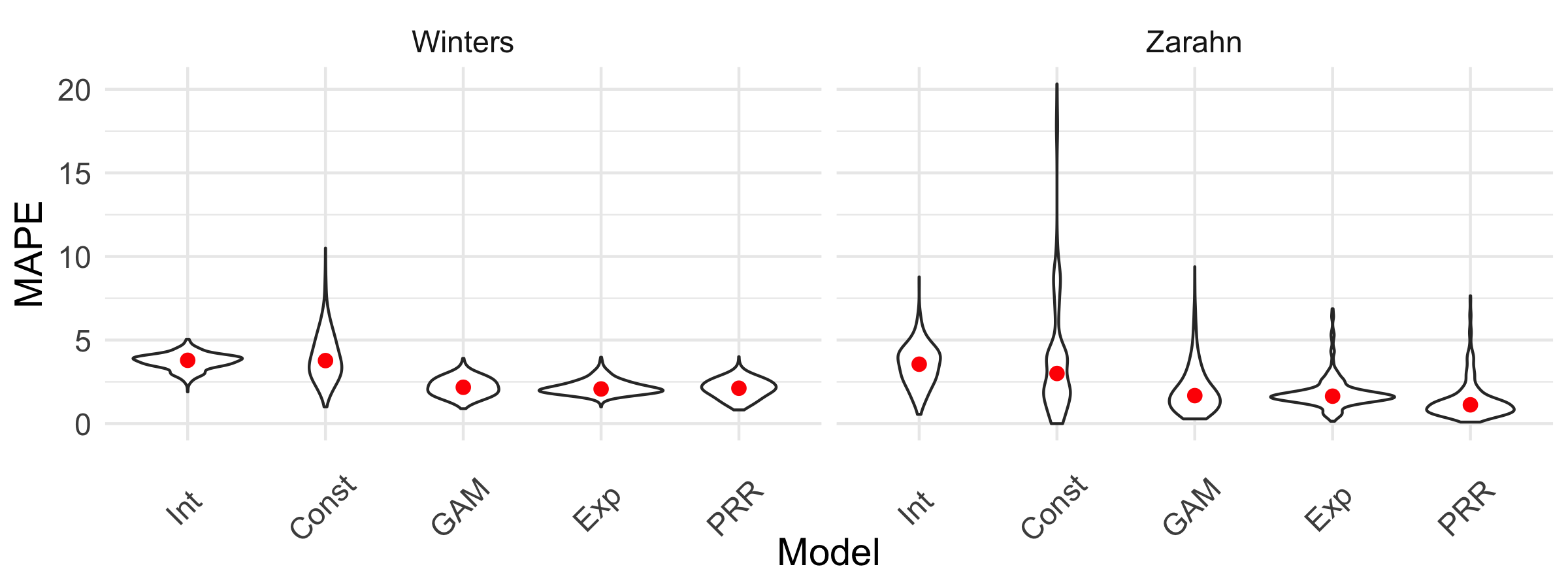

Supplement: Source data 1. [file elife-80458-data1.zip › prr_reproducibility/results/cross_valid_mape.png]

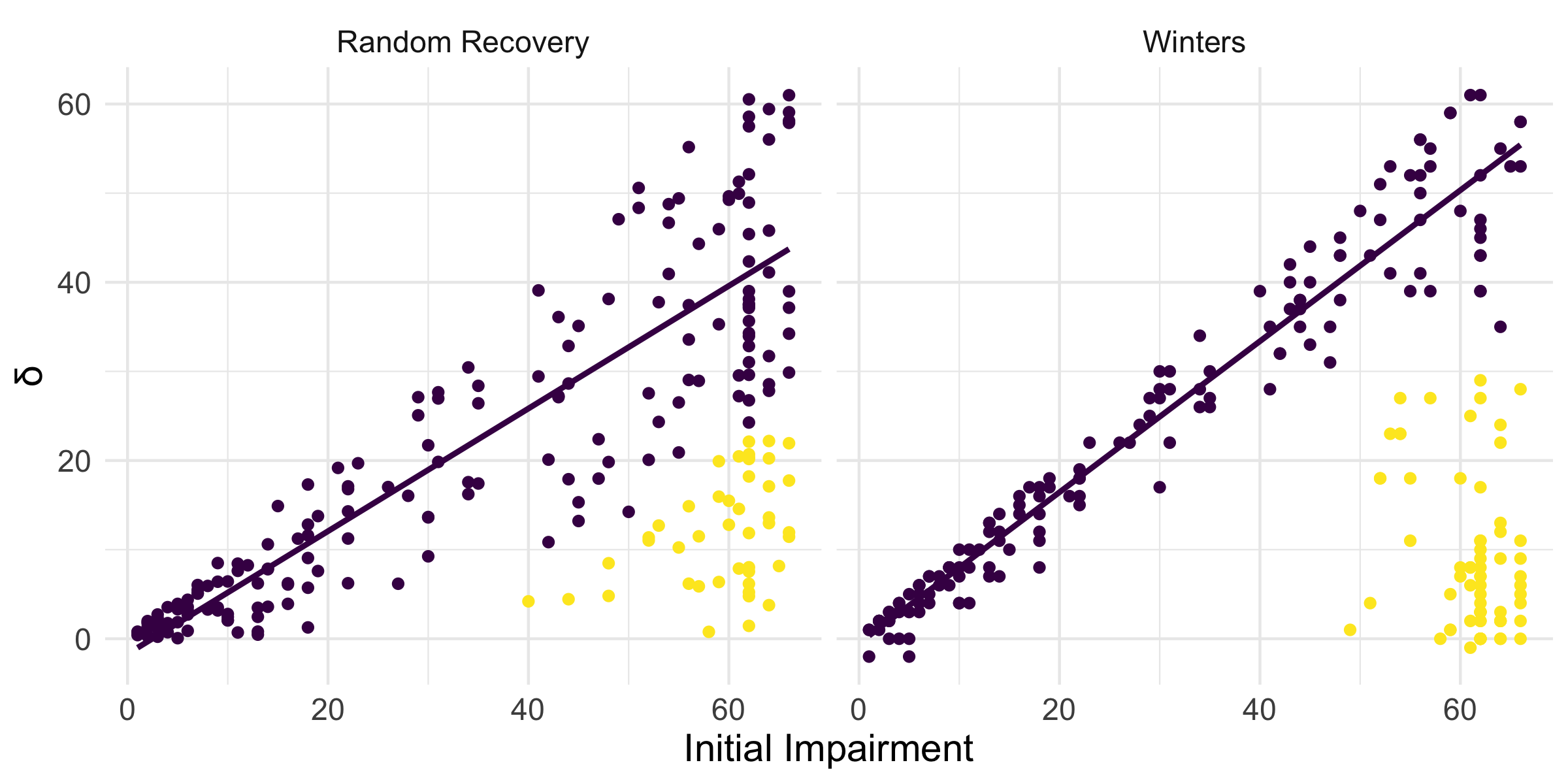

Supplement: Source data 1. [file elife-80458-data1.zip › prr_reproducibility/results/postcluster.png]

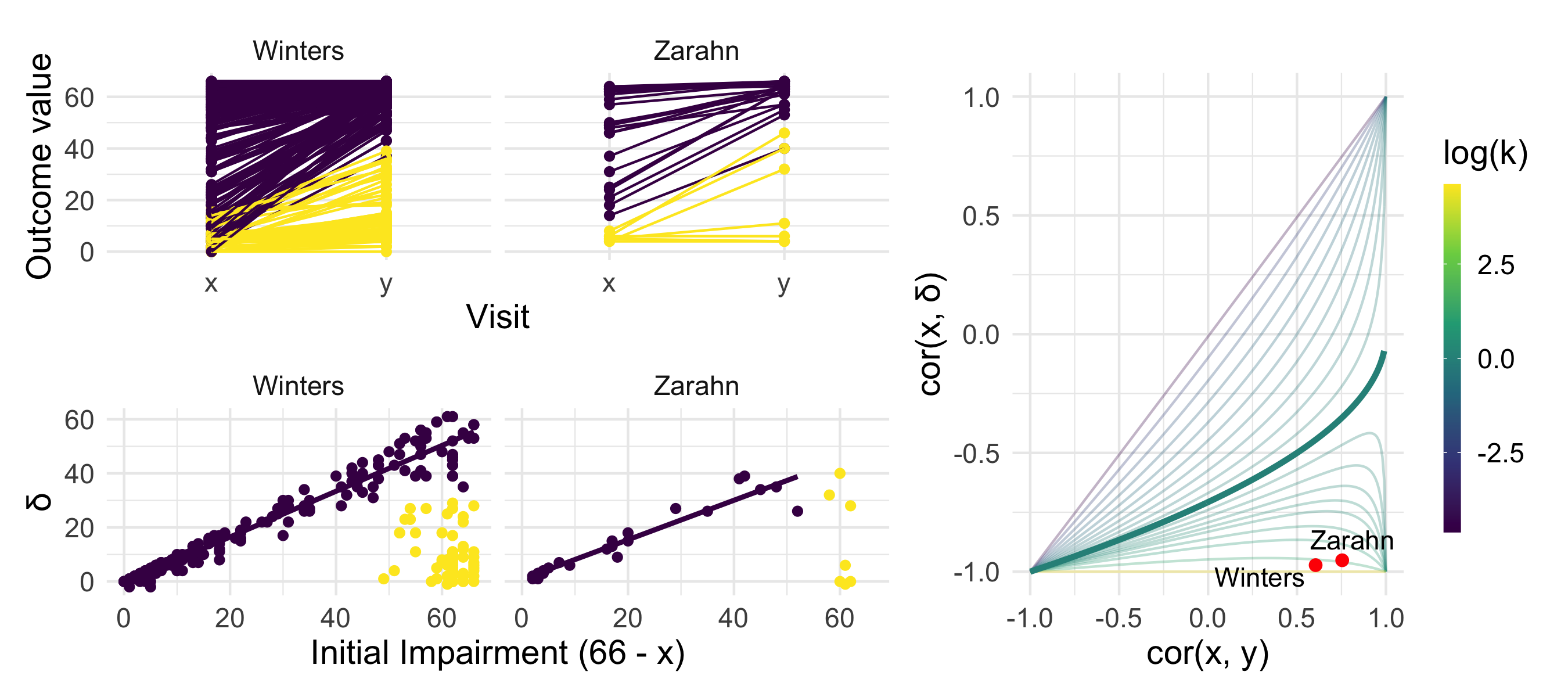

Supplement: Source data 1. [file elife-80458-data1.zip › prr_reproducibility/results/reported_data.png]

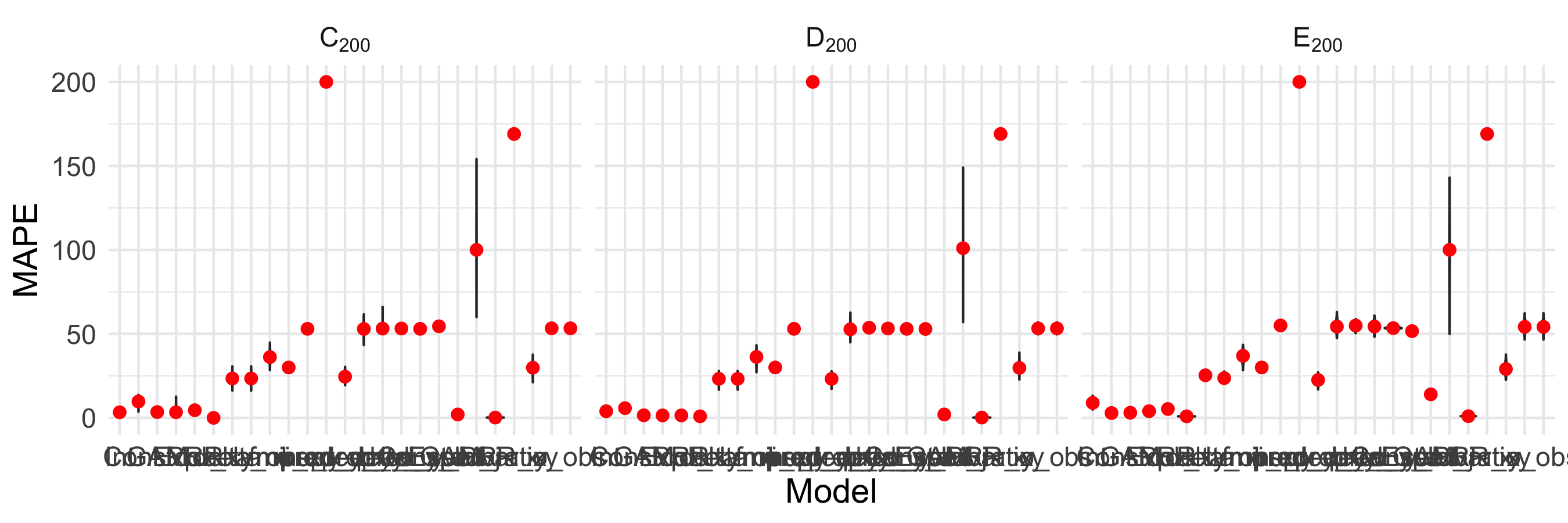

Supplement: Source data 1. [file elife-80458-data1.zip › prr_reproducibility/results/compare_model_cv.png]

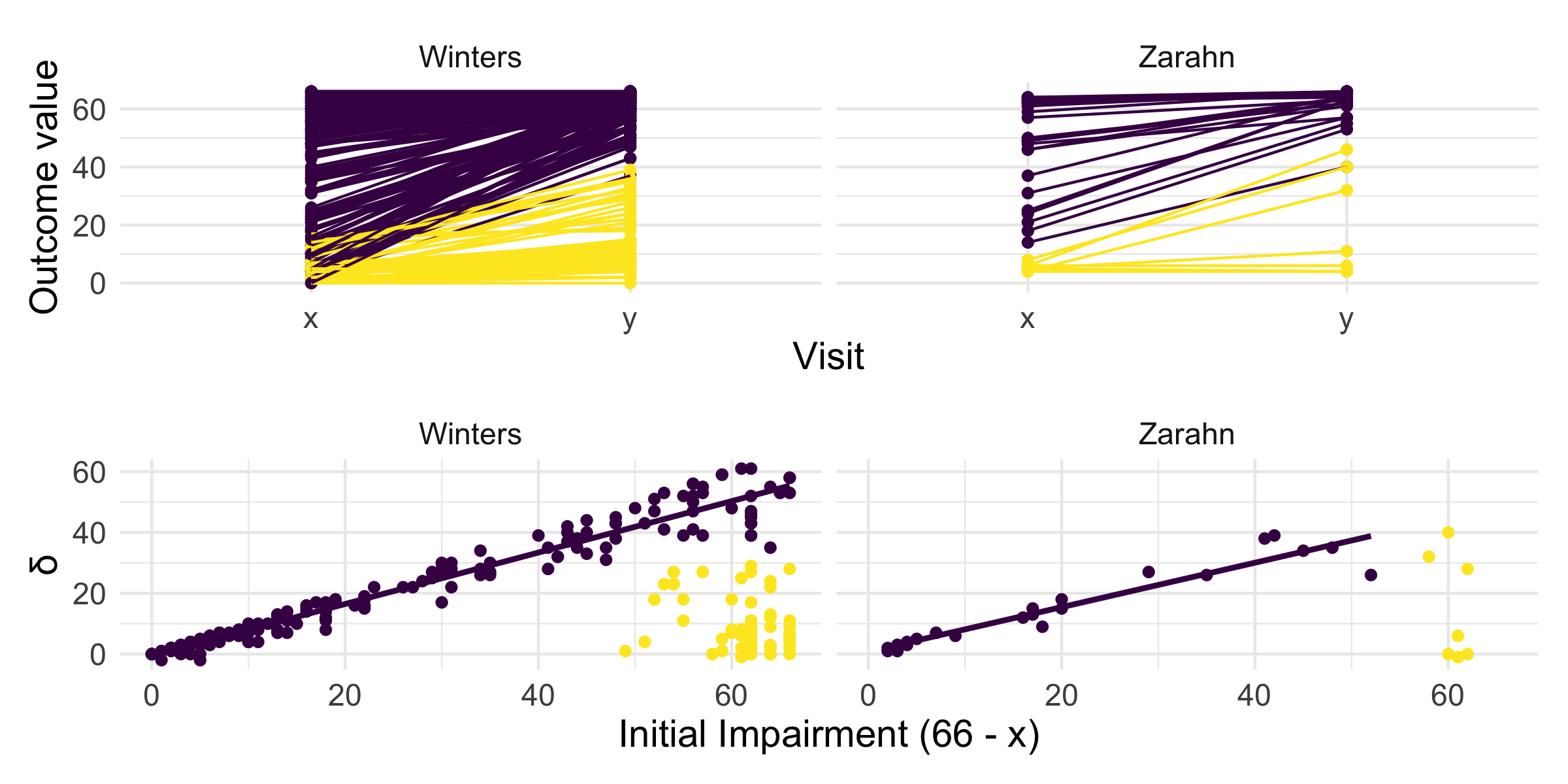

Supplement: Source data 1. [file elife-80458-data1.zip › prr_reproducibility/results/regression.png]

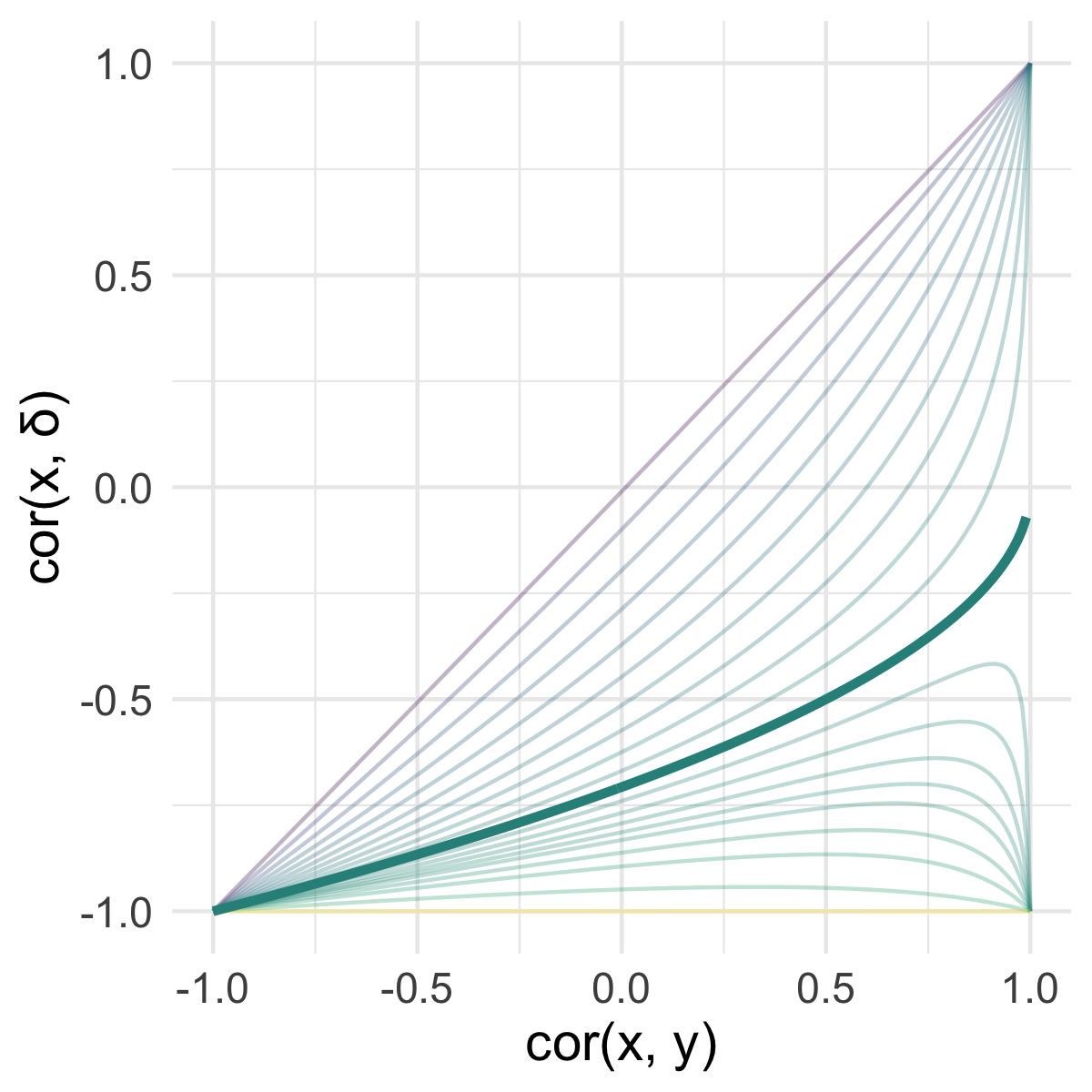

Supplement: Source data 1. [file elife-80458-data1.zip › prr_reproducibility/results/cor_plot_base.png]

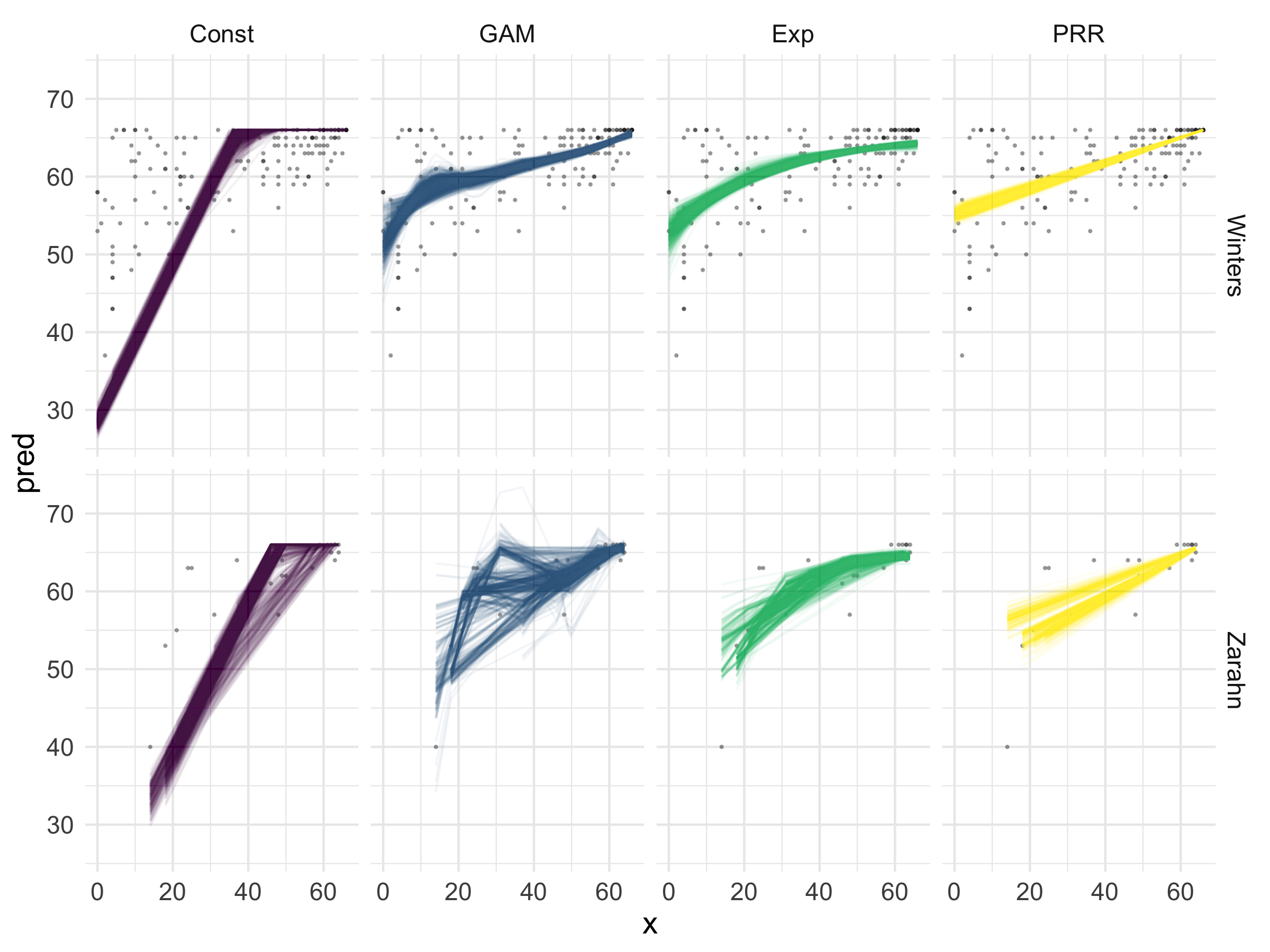

Supplement: Source data 1. [file elife-80458-data1.zip › prr_reproducibility/results/cross_valid_fits.png]

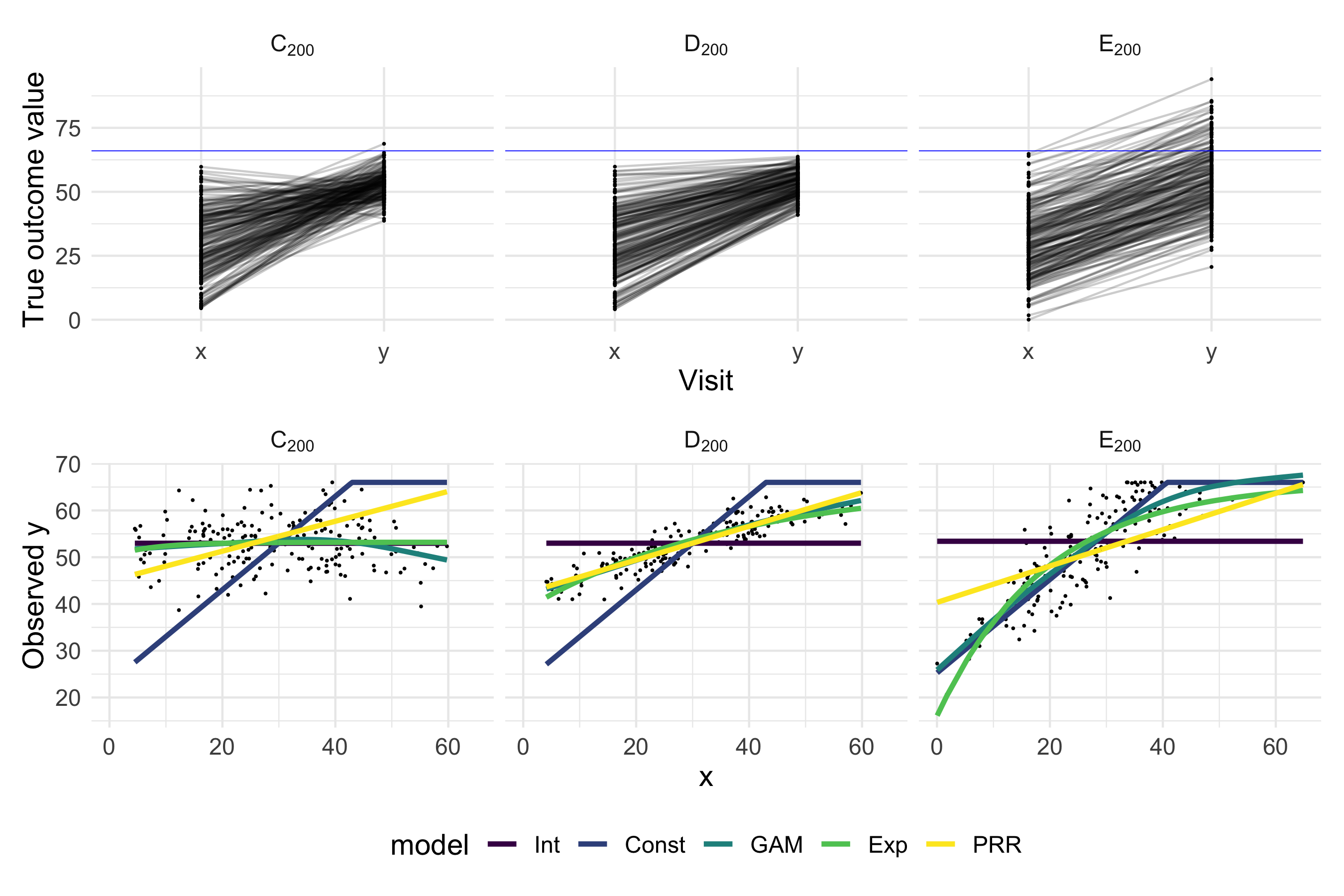

Supplement: Source data 1. [file elife-80458-data1.zip › prr_reproducibility/results/compare_model.png]

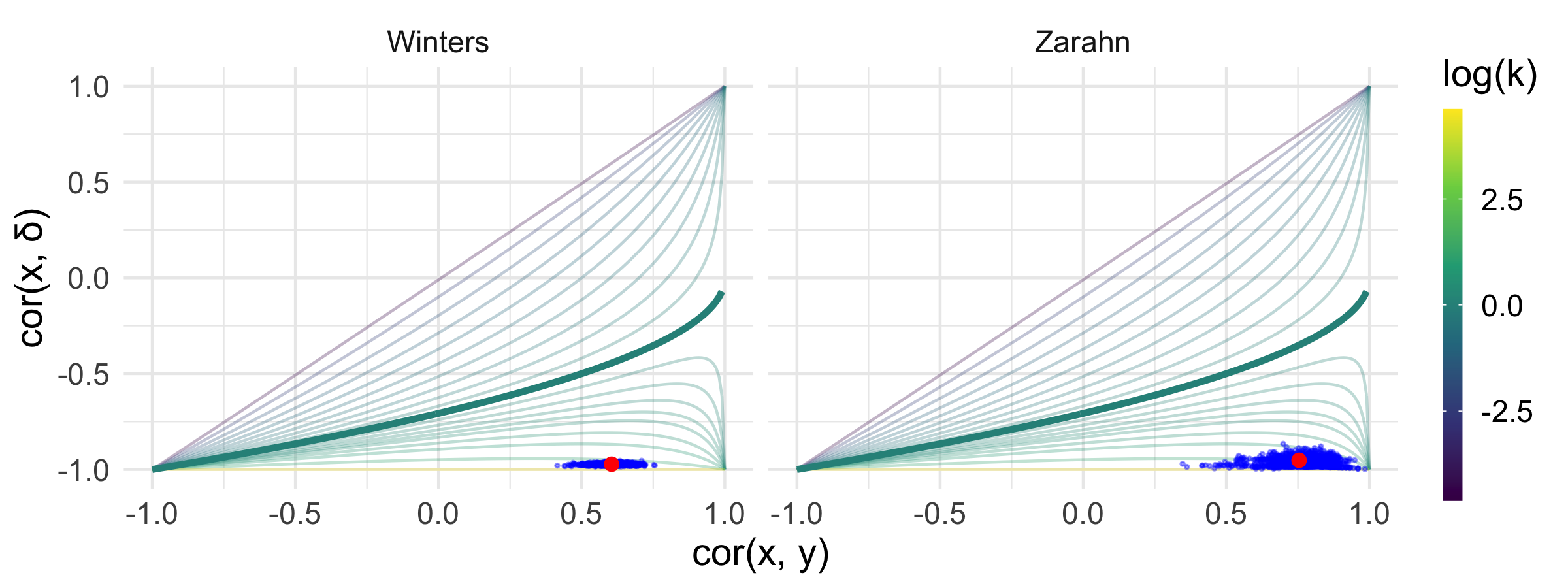

Supplement: Source data 1. [file elife-80458-data1.zip › prr_reproducibility/results/boot_analysis.png]
